# Infant Meningococcal Vaccination: Advisory Committee on Immunization Practices (ACIP) Recommendations and Rationale

**Published:** 2013-01-25

**Authors:** Lorry Rubin, Amanda Cohn, Jessica MacNeil, Nancy Messonnier

**Affiliations:** Meningococcal Vaccines Work Group, Advisory Committee on Immunization Practices, Atlanta, GA; Div of Bacterial Diseases, National Center for Immunization and Respiratory Diseases, CDC

At its October 2012 meeting, the Advisory Committee on Immunization Practices (ACIP) voted to recommend vaccination against meningococcal serogroups C and Y for children aged 6 weeks through 18 months at increased risk for meningococcal disease. Meningococcal groups C and Y and *Haemophilus* b tetanus toxoid conjugate vaccine (Hib-MenCY-TT [MenHibrix, GlaxoSmithKline Biologicals]) is licensed for active immunization for prevention of invasive disease caused by *Haemophilus influenzae* type b (Hib) and meningococcal serogroups C and Y. Hib-MenCY-TT is not indicated for prevention of disease caused by meningococcal serogroup B, the most common serogroup causing disease in infants, or serogroups W135 or A, which are represented in quadrivalent meningococcal vaccines. Before licensure of Hib-MenCY-TT, no meningococcal conjugate vaccine was licensed for infants aged 2 through 8 months. MenACWY-D (Menactra, Sanofi Pasteur) is licensed as a 2-dose series for infants and toddlers aged 9 through 23 months, and MenACWY-D and MenACWY-CRM (Menveo, Novartis Vaccines) are licensed for persons aged 2 through 55 years as a single dose. These vaccines are recommended routinely for persons aged 11 through 18 years and persons aged 2 through 55 years at increased risk for meningococcal disease (and persons aged 9 months through 55 years for MenACWY-D) (1,2). This report summarizes the deliberations of ACIP, the rationale for its decision, and recommendations for use of Hib-MenCY-TT in infants at increased risk for meningococcal disease.

## Methods

On June 14, 2012, the Food and Drug Administration licensed Hib-MenCY-TT for the prevention of invasive Hib and serogroups C and Y meningococcal disease in children aged 6 weeks through 18 months (3). In monthly teleconferences during 2009–2012 and annual in-person meetings, ACIP’s Meningococcal Vaccines Work Group reviewed safety and immunogenicity data from phase 2 and phase 3 clinical trials as well as data on disease epidemiology and the infant vaccination schedule. The work group reviewed published peer-reviewed literature and unpublished data that were relevant to infant meningococcal vaccination. Summaries of the data that were reviewed and work group discussions were presented to ACIP before recommendations were proposed. Proposed infant meningococcal vaccination recommendations were presented at the October 2012 ACIP meeting and approved by ACIP.

## Hib-MenCY-TT Safety and Immunogenicity

Hib-MenCY-TT is a combination of three discrete polysaccharide-protein conjugates. Each capsular polysaccharide is bound covalently to tetanus toxoid. The first dose may be given as early as age 6 weeks. The fourth dose may be given as late as age 18 months. Hib-MenCY-TT is supplied as a single-dose vial of lyophilized vaccine to be reconstituted with the accompanying vial of saline diluent (3). Hib-MenCY-TT effectiveness was inferred based on the following: 1) Hib antibody responses after Hib-MenCY-TT vaccination that were comparable to antibody responses after Hib-TT (first 3 doses) or Hib polyribosylribitol phosphate–meningococcal outer membrane protein (PRP-OMP) (PedvaxHIB, Merck and Co) (fourth dose) vaccination, and 2) the proportion of persons with measurable meningococcal serogroups C and Y serum bactericidal activity using human complement (hSBA) after Hib-MenCY-TT vaccination. Evaluation of hSBA responses in clinical studies could be used to infer protection because an association between serum bactericidal activity and clinical effectiveness already exists (4). In the United States, meningococcal clinical endpoint efficacy trials with Hib-MenCY-TT were not feasible, and no meningococcal vaccine is licensed and available for this age group to allow a comparative trial.

A single-blinded, controlled, multicenter study with two parallel randomized groups was conducted to evaluate safety and immunogenicity of Hib-MenCY-TT compared with U.S.-licensed Hib-TT (ActHIB, Sanofi Pasteur) in healthy infants at ages 2, 4, and 6 months (5). The proportions of children who, after dose 3, had hSBA titers ≥1:8 (the clinical threshold defined as protective) to serogroups C and Y were 99% and 96%, respectively (5). The proportion of children who had anti-HibPRP antibody concentrations ≥1.0 *μ*g/mL (the accepted level indicative of long-term protection) after dose 3 was 96% in the Hib-MenCY-TT group and 91% in the Hib-TT group (5). Hib-MenCY-TT also was evaluated before and after the fourth dose given at age 12–15 months. HibPRP-OMP was used in the control vaccine group. The proportion of subjects with hSBA titers ≥1:8 was 99% for serogroups C and Y 1 month after the fourth dose. The Hib response after the fourth dose also was demonstrated to be noninferior to HibPRP-OMP (the percentage of subjects with anti-HibPRP antibody concentrations ≥1.0 *μ*g/mL was 99.2% in both treatment groups).

Hib-MenCY-TT was co-administered with DTaP-HepB-IPV and 7-valent pneumococcal conjugate vaccine (PCV7) at ages 2, 4, and 6 months, and with measles-mumps-rubella, varicella, and PCV7 vaccines at age 12–15 months. In clinical trials, no decreased immunogenicity of coadministered vaccines was observed (5,6). A randomized, controlled, multicenter study evaluated the percentage of subjects with hSBA titers ≥1:8 at 2 months after the second dose was administered at age 4 months. In the group vaccinated with Hib-MenCY-TT, 94% and 83% of subjects achieved hSBA antibody titers ≥1:8 for meningococcal serogroups C and Y, respectively, after dose 2 (7). Rates of local and systemic adverse events observed after administration of Hib-MenCY-TT were comparable to rates observed after administration of Hib-TT. Thus, Hib-MenCY-TT was found to be safe and immunogenic for both Hib and meningococcal serogroups C and Y.

## Summary of ACIP Deliberations and Rationale

### Infants at increased risk for meningococcal disease

Infants with persistent complement component pathway deficiencies or functional or anatomical asplenia have an increased risk for meningococcal disease compared with healthy infants. Complement component deficiencies rarely are observed in infancy, but infants might be identified because of family history. Certain infants with complex congenital heart disease have asplenia, and infants with sickle cell disease often are identified via newborn screening programs. Infants with sickle cell disease initially might have functioning spleens, but develop functional asplenia during early childhood. Infrequently, healthy infants also might be at increased risk because of a serogroups C or Y meningococcal disease outbreak for which vaccination is recommended. The number of U.S. infants in these high-risk groups is small (estimated at 3,000–5,000), making a targeted high-risk vaccination policy feasible and reasonable given the potential increased risk in these infants. Infants who are traveling with their families to the Hajj or to the “meningitis belt” of sub-Saharan Africa need protection against serogroups A and W135, which are not in Hib-MenCY-TT, and should receive a quadrivalent meningococcal conjugate vaccination licensed for children aged ≥9 months before travel (8).

### Infants not at increased risk for meningococcal disease

ACIP reviewed the burden of meningococcal disease among infants and children aged 0–59 months. Meningococcal disease is a serious, but rare, infectious disease. Rates of meningococcal disease have declined in all age groups since 2000, and, in 2011, the overall rate of meningococcal disease was at a historic low of 0.21 per 100,000 population (CDC, unpublished data, 2011). In the United States, during 1993–2011, average annual rates of meningococcal disease were higher among children aged 0 through 59 months (1.74 per 100,000 population) than in adolescents aged 11 through 19 years (0.57 per 100,000) (CDC, unpublished data, 2011). However, approximately 60% of disease among children aged 0 through 59 months is caused by serogroup B meningococcal disease, which is not prevented by any meningococcal vaccine licensed in the United States. Additionally, the highest incidence in the first 5 years of life occurs in infants aged 0 through 6 months, most of whom are too young to have received the minimum 2 or 3 doses of vaccine that likely will be needed to provide protection. The case-fatality ratio of meningococcal disease caused by serogroups C and Y is lower among children aged <59 months (6%) compared with adolescents (11%) (9,10). During 2007–2009, approximately 77 cases and four to eight deaths from serogroups C and Y *Neisseria meningitidis* occurred annually in children aged <59 months. For the estimated 205 annual cases of meningococcal disease in children aged <59 months that occurred during 2007–2009, a universal infant meningococcal vaccination program would have prevented 40–50 cases (nearly 25% of cases in this age group) (CDC, unpublished data, 2012). The epidemiology of meningococcal disease is dynamic, and rates of disease could increase in the future, requiring a reassessment of immunization strategy.

Presentations, including 1) a cost-effectiveness analysis of vaccinating all U.S. infants, 2) programmatic aspects of adding meningococcal vaccination to the infant routine immunization schedule, and 3) results of a survey evaluating attitudes of pediatricians and family physicians toward vaccinating all infants with meningococcal vaccines, were made at the October 2011 ACIP meeting and summarized during the October 2012 ACIP meeting.* These considerations support the ACIP decision, but the current epidemiology of meningococcal disease is the primary rationale for the decision. In summary, the current low burden of disease, as well as the low proportion of meningococcal cases that are preventable with vaccines that do not protect against serogroup B disease, limit the potential impact of a routine meningococcal vaccination program in infants in the United States. Therefore, ACIP concluded that a targeted approach to protect infants at increased risk for meningococcal disease was the optimal vaccination strategy at this time. At the October 2012 ACIP meeting, ACIP voted to recommend vaccination with Hib-MenCY-TT only for infants at increased risk for meningococcal disease.

## ACIP Recommendations for Infants at Increased Risk for Meningococcal Disease

Infants at increased risk for meningococcal disease should be vaccinated with a 4-dose series of Hib-MenCY-TT. These include infants with recognized persistent complement pathway deficiencies and infants who have anatomic or functional asplenia including sickle cell disease. Additionally, Hib-MenCY-TT can be used in infants aged 6 weeks through 18 months who are in communities with serogroups C and Y meningococcal disease outbreaks, but Hib-MenCY-TT is not adequate for infants traveling to the Hajj or the “meningitis belt” of sub-Saharan Africa (a quadrivalent meningococcal vaccine that contains serogroups A and W135 is required for those infants and may be given starting at age 9 months).

If an infant at increased risk for meningococcal disease is behind on his or her Hib vaccine doses, Hib-MenCY-TT may be used following the same catch-up schedule used for Hib vaccine. However, if the first dose of Hib-MenCY-TT is given at or after 12 months of life, 2 doses should be given at least 8 weeks apart to ensure protection against serogroups C and Y meningococcal disease. For infants at increased risk for meningococcal disease who have received or are going to receive a different Hib vaccine product, ACIP recommends a 2-dose series of MenACWY-D if they are aged 9 through 23 months or either of the two quadrivalent meningococcal vaccine products after age 23 months.

Hib-MenCY-TT may be co-administered with other routine infant vaccinations, including 13-valent pneumococcal conjugate vaccine. Hib-MenCY-TT should not be co-administered with other Hib-containing vaccines.

## Guidance for Use of Hib-MenCY-TT

Based on an assessment of the potential public health impact, including the current low incidence of meningococcal disease in the United States, at this time ACIP does not recommend routine meningococcal vaccination for infants who are not at increased risk for meningococcal disease. Hib-MenCY-TT is safe and immunogenic against Hib and *N. meningitidis* serogroups C and Y. Hib-MenCY-TT may be used in any infant for routine vaccination against Hib and will offer some protection against serogroups C and Y meningococcal disease. Four doses of Hib-MenCY-TT fulfill the primary series and booster dose Hib immunization recommendations. If Hib-MenCY-TT vaccine is used to achieve protection against serogroups C and Y, Hib-MenCY-TT should be used for all 4 doses of Hib vaccine. Because the protection offered by meningococcal vaccines wanes over time, an infant series will be unlikely to provide persistent protection against meningococcal disease until age 11–12 years, the age of recommended adolescent vaccination. Infants and children who received Hib-MenCY-TT and are travelling to areas with high endemic rates of meningococcal disease such as the “meningitis belt” are not protected against serogroups A and W-135 and should receive a quadrivalent meningococcal conjugate vaccine licensed for children aged ≥9 months before travel.

ACIP will continue to reevaluate trends in epidemiology to determine whether meningococcal vaccines should be added to the routine infant schedule and what schedule should be implemented for reimmunization. Vaccines that provide long-term protection against meningococcal disease early in life have the potential to reduce the burden of meningococcal disease, especially if they provide protection against serogroup B meningococcal disease. Health-care providers should be aware of the continued need for early recognition and treatment of meningococcal disease.†

## Footnotes

* Additional information available at http://www.cdc.gov/vaccines/acip/meetings/meetings-info.html.

† Information available at http://www.cdc.gov/meningitis.
